# Tuberculosis incidence in foreign-born people residing in European countries in 2020

**DOI:** 10.2807/1560-7917.ES.2023.28.42.2300051

**Published:** 2023-10-19

**Authors:** Anca Vasiliu, Niklas Köhler, Ekkehardt Altpeter, Tinna Rán Ægisdóttir, Marina Amerali, Wouter Arrazola de Oñate, Ágnes Bakos, Stefania D’Amato, Daniela Maria Cirillo, Reinout van Crevel, Edita Davidaviciene, Irène Demuth, Jose Domínguez, Raquel Duarte, Gunar Günther, Jean-Paul Guthmann, Sophia Hatzianastasiou, Louise Hedevang Holm, Zaida Herrador, Urška Hribar, Conny Huberty, Elmira Ibraim, Sarah Jackson, Mogens Jensenius, Kamilla Sigridur Josefsdottir, Anders Koch, Maria Korzeniewska-Kosela, Liga Kuksa, Heinke Kunst, Christian Lienhardt, Beatrice Mahler, Mateja Janković Makek, Inge Muylle, Johan Normark, Analita Pace-Asciak, Goranka Petrović, Despo Pieridou, Giulia Russo, Olena Rzhepishevska, Helmut J.F. Salzer, Marta Sá Marques, Daniela Schmid, Ivan Solovic, Mariya Sukholytka, Petra Svetina, Mariya Tyufekchieva, Tuula Vasankari, Piret Viiklepp, Kersti Villand, Jiri Wallenfels, Stefan Wesolowski, Anna-Maria Mandalakas, Leonardo Martinez, Dominik Zenner, Christoph Lange, on behalf of the TBnet

**Affiliations:** 1 Baylor College of Medicine, Department of Pediatrics, Global and Immigrant Health, Global Tuberculosis Program, Houston, Texas, United States; 2 Division of Clinical Infectious Diseases, Research Center Borstel, Borstel, Germany; 3 German Center for Infection Research (DZIF), TTU-TB, Borstel, Germany; 4 Respiratory Medicine & International Health, University of Lübeck, Lübeck, Germany; 5 Swiss Federal Office of Public Health, Division of Communicable Diseases, Bern, Switzerland; 6 The National University Hospital of Iceland, Pharmaceutical Services, Reykjavik, Iceland; 7 Tuberculosis Control Office, Department of Respiratory Infections, Directorate for Epidemiological Surveillance & Intervention, National Public Health Organization (NPHO), Athens, Greece; 8 Belgian Lung and Tuberculosis Association, Brussels, Belgium; 9 Flemish Association of Respiratory Health and TB Control, Leuven, Belgium; 10 Koranyi National Institute for Pulmonology, Budapest, Hungary; 11 Prevention of Communicable Diseases and International Prophylaxis, General Direction of Health Prevention, Ministry of Health of Italy, Rome, Italy; 12 Emerging Bacterial Pathogens Unit, IRCCS San Raffaele Scientific Institute, Milan, Italy; 13 Department of Internal Medicine and Radboud Center for Infectious Diseases, Radboud University Medical Center, Nijmegen, the Netherlands; 14 Centre for Tropical Medicine and Global Health, Nuffield Department of Medicine, University of Oxford, Oxford, United Kingdom; 15 Vilnius University hospital Santaros Klinikos, Department of Tuberculosis State information system, Vilnius, Lithuania; 16 Health Directorate of Luxembourg, Luxembourg; 17 Institut d’Investigació Germans Trias i Pujol; Universitat Autònoma de Barcelona; CIBER Enfermedades Respiratorias; INNOVA4TB consortium Badalona, Barcelona, Spain; 18 ICBAS – Instituto de Ciências Biomédicas Abel Salazar, Universidade do Porto; 19 ISPUP – Instituto de Saúde Pública da Universidade do Porto, Porto, Portugal; 20 Centro Hospitalar de Vila Nova de Gaia/Espinho, Porto, Portugal; 21 Department of Pulmonary Medicine and Allergology, Inselspital, Bern University Hospital, University of Bern, Switzerland; 22 Department of Medical Sciences, School of Medicine, University of Namibia, Windhoek, Namibia; 23 Division of Infectious Diseases, Santé publique France, Saint-Maurice, France; 24 Department of Infectious Disease Epidemiology and Prevention, Statens Serum Institut, Copenhagen, Denmark; 25 Centro Nacional de Epidemiología, Instituto de Salud Carlos III, Madrid, Spain; 26 Tuberculosis Register of the Republic of Slovenia, University Clinic Golnik, Golnik, Slovenia; 27 Marius Nasta Institute of Pulmonology, Bucharest, Romania; 28 Infectious Diseases; Health Service Executive Health Protection Surveillance Centre, Dublin, Ireland; 29 Department of Infectious Diseases, Oslo University Hospital, Ullevaal, Norway; 30 Centre for Health Security and Communicable Disease Control, Directorate of Health, Iceland; 31 Department of Infectious Diseases, Rigshospitalet University Hospital, Copenhagen, Denmark; 32 Department of Tuberculosis Epidemiology and Surveillance, National Tuberculosis and Lung Diseases Research Institute, Warsaw, Poland; 33 Riga East University Hospital, TB and Lung Disease Clinic, Riga, Latvia; 34 Blizard Institute, The London School of Medicine and Dentistry, Queen Mary University of London, London, United Kingdom; 35 Unité Unité Mixte Internationale 233 IRD – U1175 INSERM - Université de Montpellier, Institut de Recherche pour le Développement (IRD), Montpellier, France; 36 Epidemiology and Population Health, Department of Infectious Disease Epidemiology, London School of Hygiene and Tropical Medicine, London, United Kingdom; 37 Department Cardio-thoracic, Pneumophtisiology II, University of Medicine and Pharmacy “Carol Davila” Bucharest, Romania; 38 University of Zagreb, School of Medicine Zagreb, Croatia; 39 University Hospital Centre Zagreb, Department for Lung diseases, Zagreb, Croatia; 40 Division of Pneumology, Onze-Lieve-Vrouw Ziekenhuis (OLV) Aalst, Aalst, Belgium; 41 Department of Clinical Microbiology, Umeå University, Sweden; 42 Wallenberg Centre for Molecular Medicine, Umeå University, Sweden; 43 Infectious Disease Prevention and Control Unit, Health Promotion and Disease Prevention Directorate, Superintendence of Public Health, Ministry for Health of Malta, La Valetta, Malta; 44 Respiratory Diseases and Travel Medicine Department with Vaccination Unit, Infectious Diseases Epidemiology ServiceDepartment, Croatian Institute of Public Health, Zagreb, Croatia; 45 Cyprus National Reference Laboratory for Mycobacteria, Microbiology Department, Nicosia General Hospital, Nicosia, Cyprus; 46 Department of Chemistry, Department of Clinical Microbiology, Umeå University, Sweden; 47 Division of Infectious Diseases and Tropical Medicine, Department of Internal Medicine 4- Pneumology, Kepler University Hospital, Linz, Austria; 48 Faculty of Medicine, Johannes-Kepler-University, Linz, Austria and Ignaz Semmelweis Institut, Interuniversity Institute for Infection Resarch, Vienna, Austria; 49 Unit for Infectious Diseases Diagnostics and Infectious Diseases Epidemiology, Centre for Pathophysiology, Infectious Diseases and Immunology, Medical University of Vienna, Vienna, Austria; 50 National Institute for TB, Lung Diseases and Thoracic Surgery, Vysne Hagy, Slovakia; 51 Catholic University Ruzomberok, Ruzomberok, Slovakia; 52 First Faculty of Medicine and Faculty Thomayer Hospital Prague, Czechia; 53 National TB Program and Tuberculosis Registry of Republic of Slovenia, University Clinic of Respiratory and Allergic Diseases Golnik, Golnik, Slovenia; 54 Health Promotion and Prevention Unit, Directorate Public Health Protection and Health Control, Ministry of Health of Bulgaria, Sofia, Bulgaria; 55 University of Turku, Division of Medicine, Department of Pulmonary Diseases and Clinical Allergology, Turku, Finland; 56 Finnish Lung Health Association (Filha ry), Helsinki, Finland; 57 Estonian Tuberculosis Register, Dept. of Registries, National Institute for Health Development, Tallinn, Estonia; 58 National TB Surveillance Unit, University Hospital Bulovka, Prague, Czechia; 59 Boston University, School of Public Health, Department of Epidemiology, Boston, Massachusetts, United States; 60 Global Public Health Unit, Wolfson Institute of Population Health Barts; 61 The London School of Medicine and Dentistry, Queen Mary University of London, London, United Kingdom; 62 The Tuberculosis Network European Trials Group (TBNET) (www.tbnet.eu)

**Keywords:** migrants, policy, prevention, refugees, TB

## Abstract

**Background:**

European-specific policies for tuberculosis (TB) elimination require identification of key populations that benefit from TB screening.

**Aim:**

We aimed to identify groups of foreign-born individuals residing in European countries that benefit most from targeted TB prevention screening.

**Methods:**

The Tuberculosis Network European Trials group collected, by cross-sectional survey, numbers of foreign-born TB patients residing in European Union (EU) countries, Iceland, Norway, Switzerland and the United Kingdom (UK) in 2020 from the 10 highest ranked countries of origin in terms of TB cases in each country of residence. Tuberculosis incidence rates (IRs) in countries of residence were compared with countries of origin.

**Results:**

Data on 9,116 foreign-born TB patients in 30 countries of residence were collected. Main countries of origin were Eritrea, India, Pakistan, Morocco, Romania and Somalia. Tuberculosis IRs were highest in patients of Eritrean and Somali origin in Greece and Malta (both > 1,000/100,000) and lowest among Ukrainian patients in Poland (3.6/100,000). They were mainly lower in countries of residence than countries of origin. However, IRs among Eritreans and Somalis in Greece and Malta were five times higher than in Eritrea and Somalia. Similarly, IRs among Eritreans in Germany, the Netherlands and the UK were four times higher than in Eritrea.

**Conclusions:**

Country of origin TB IR is an insufficient indicator when targeting foreign-born populations for active case finding or TB prevention policies in the countries covered here. Elimination strategies should be informed by regularly collected country-specific data to address rapidly changing epidemiology and associated risks.

**Key public health message**

**What did you want to address in this study?**

One of three patients with tuberculosis (TB) in the European Union/European Economic Area (EU/EEA) is foreign-born. However, there is limited evidence regarding risk assessment, curative and preventive management of TB in foreign-born individuals. We wanted to provide an evidence base for improved TB detection and prevention policies among foreign-born individuals in the EU/EEA.

**What have we learnt from this study?**

The burden of TB among foreign-born individuals is highly diverse and heterogeneously distributed across Europe. It often reflects historical links. A high TB incidence rate in a person’s country of origin is often not aligned with the risk of TB in their country of residence. Thus this should not be the only indicator used to inform guidelines for active case finding and TB prevention in EU/EEA countries.

**What are the implications of your findings for public health?**

The TB incidence rate in the country of origin is an insufficient indicator when targeting foreign-born populations for active case finding or TB prevention policies in Europe. Tuberculosis elimination strategies should be informed by regularly collected country-specific data to ensure that rapidly changing epidemiology and associated risks are addressed.

## Introduction

In the year 1882, when Robert Koch identified a Mycobacterium as the causative agent of tuberculosis (TB), the disease was a common and a leading cause of death in Europe [1].

Migration to European countries, in particular the European Union and the European Economic Area (EU/EEA) accounts for nearly a third of global migration, with 87 million foreign-born individuals living in Europe in 2020 [2]. Foreign-born individuals residing in low TB incidence countries have a higher risk of TB than individuals born in these low incidence countries [3]. However, reasons for the increased risk of TB in this population are poorly understood and much is still unknown about the epidemiology in this heterogenous group with regard to the routes of migration, incomes, countries of origin, etc.

The incidence of TB in the EU/EEA has steadily declined in the last century [4]. Tuberculosis is now a rare disease and a very rare cause of death in most EU/EEA countries [4]. According to the European Centre for Disease Prevention and Control (ECDC) and the World Health Organization (WHO) Regional office for Europe, the estimated TB incidence rate (IR) in the EU/EEA in 2020 was 9.5 per 100,000 population (95% confidence interval (CI): 9.1–10.0) [4], which was low compared with the global IR of 124 per 100,000 [5]. Projections regarding the declining incidence of TB in the EU/EEA suggest that elimination of TB appears to be possible before 2040 [4] as predicted by the WHO END-TB strategy [6]. Nevertheless, the decrease in notification rates in native residents is higher than in foreign-born individuals [7].

In 2020, approximately one third of TB cases (10,942/33,148) occurred in foreign-born individuals in the EU/EEA [4]. In some EU/EEA countries such as Denmark, Germany, Luxemburg, Norway, the Netherlands and Sweden, more than 60 percent of TB cases occur among foreign-born residents [4]. To achieve TB elimination in Europe, it is necessary to timely identify TB among foreign-born individuals [8].

One of the core activities to achieve elimination of TB in countries with low TB incidence is prevention [9], and this relies on active case finding and treatment, vaccination and preventive treatment [10,11]. Efforts aimed at eliminating TB should ensure that preventive measures target key populations at risk of TB [8]. These measures mainly include active case finding strategies as well as providing TB preventive treatment to exposed individuals (i.e. TB contacts and individuals recently arrived from a high TB incidence country) according to national migrant screening programmes [10].

A programmatic management guideline for latent infection with *Mycobacterium tuberculosis* (LTBI) by ECDC suggests that people coming from high TB incidence countries should be considered for LTBI screening [12]. Interventions should be based on the epidemiological situation of TB in the country of arrival, TB incidence in the country of origin as well as on the travel route, reason for migration and time since leaving the country of origin. However, so far, the relationship between the incidence of TB in the country of origin and the risk of TB in foreign-born individuals in low TB incidence countries in Europe is ill-defined. Further, limited evidence prevents consensus regarding risk assessment and preventive management of TB in foreign-born individuals.

To provide an evidence base for TB detection and prevention policies, we analysed the incidence of TB among foreign-born individuals in EU countries and Iceland, Norway, Switzerland and the UK. We hypothesise that TB incidence rates among foreign-born residents in the EU and Iceland, Norway, Switzerland and the UK reflect TB incidence rates in their countries of origin.

## Methods

We conducted a multinational cross-sectional survey among the members of the Tuberculosis Network European Trials group (TBnet) to gather information on foreign-born individuals with TB (some countries, e.g. Germany, provide data on nationality only), indirectly indicating the flow of migrants to the European region. Country representatives from all 27 countries of the European Union as well as Iceland, Norway, Switzerland and the UK were contacted by the study coordinators between May and September 2022 and asked to provide data via a standardised questionnaire. We defined the country of origin as the country where an individual was born and migrated from. We defined the country of residence as the country in which individuals with TB were notified, i.e. the countries participating in our survey. Every country of residence contributed data on (i) the 10 highest ranked countries of origin in terms of number of notified TB cases among foreign-born individuals in the year 2020; (ii) the number of foreign-born individuals with TB from these countries of origin; (iii) the total number of individuals born in these countries of origin and living in the country of residence at the end of the year 2020.

The incidence rate (IR) of TB in foreign-born individuals in the country of residence was calculated by dividing the number of new TB cases notified in the country of residence in 2020 by the number of individuals registered as not born in that country but living there at the end of 2020 (as per 100,000 population).

We considered all foreign-born individuals stemming from one country of origin and living in one country of residence as one population. Foreign-born populations were excluded from the analysis of IR and incidence rate ratio (IRR, see below) if there were fewer than 20 TB cases per country of origin or fewer than 100 foreign-born individuals per country of origin in the dataset. Countries with an IR of less than 100 per 100,000 population were considered countries of low TB burden. Tuberculosis IRs in the country of residence were compared with WHO-estimated TB IRs and IRs based on notifications in the country of origin as reported by the WHO [5]. The incidence rate ratio (IRR) of foreign-born populations was calculated by dividing the IR in the country of residence by the WHO-estimated TB IRs and IRs based on notifications in the country of origin in 2020.

Since in EU countries, as well as Iceland, Norway, Switzerland and the UK, patients with TB are likely being detected, the gap between the number of notified cases and the true number of cases is small and will be substantially smaller than the gap between the number of notified and true incident cases in most countries of origin.

We used a Poisson random effects model to estimate the relative risk (RR) of TB by country of residence and country of origin. We performed spatial analysis using Bayesian inference with integrated Laplace approximation to calculate the mean and standard deviation of the posterior densities per country of residence and country of origin. The 95% credibility interval (CrI) was calculated using the highest posterior densities.

Figures were created using GraphPad Prism version 9.4.1 (GraphPad Software, San Diego, United States), DataWrapper [13] and SankeyMATIC [14]. Data analysis was done using R version 4.3.1 (R Foundation for Statistical Computing, Vienna, Austria).

The study protocol was reviewed by independent reviewers and endorsed by the TBnet steering committee.

Reporting followed the STROBE guidelines [15].

## Results

Questionnaires were returned by the end of September 2022 for 30 of 31 countries. Data on foreign-born individuals who developed TB in 2020 in Latvia were not available as they were not recorded. Data on the number of foreign-born individuals living in Belgium, Malta and Romania were only partially available, and as data from 2020 were not available in France, we used the latest available data on the number of foreign-born individuals living in France in 2018. Malta and Greece reported low confidence in their total number of foreign-born individuals from Eritrea and Somalia. The complete dataset is shown in Supplementary Table S1. For Cyprus, Ireland and Romania there was no clear ranking for the 10th country due to an equivalent number of TB cases. Therefore, more than 10 countries were included in the analysis (Supplementary Table S2).

Country-specific sources of data on the number of foreign-born patients with TB are displayed in Supplementary Table S3.

Data describing 9,116 foreign-born TB cases were gathered via this survey (Table). These cases stemmed from 75 countries of origin and were recorded in 30 countries of residence, accounting for a total of 267 populations of foreign-born individuals.

**Table:** Number of tuberculosis cases among foreign-born residents and total number of foreign-born residents from the 10 highest ranked countries of origin in terms of the highest tuberculosis burden, European Union countries and Iceland, Norway, Switzerland and the United Kingdom, 2020

Country	TB cases among foreign-born residents^a^	Total number of foreign-born residents^a^	TB IR in foreign-born residents^a^	TB IR as reported to the WHO in 2020^b^ [5]
United Kingdom	1,997	2,579,000	77.4	6.9
Germany	1,543	4,452,141	34.7	5.5
France	1,439	3,146,068	45.7	8.2
Italy	884	2,328,403	38.0	6.6
Spain	819	2,673,525	30.6	7.3
Belgium	361	NA	NA	7.7
Netherlands	299	630,455	47.4	4.1
Portugal	267	428,734	62.3	16.0
Greece	191	732,968	26.1	4.5
Austria	169	589,765	28.7	4.9
Sweden	165	580,798	28.4	3.6
Switzerland	160	498,680	32.1	4.7
Malta	116	NA	NA	36.0
Denmark	114	143,226	79.6	4.9
Czechia	104	430,437	24.2	3.9
Ireland	96	165,080	58.2	5.3
Norway	95	165,727	57.3	3.1
Poland	81	1,564,051	5.2	9.6
Estonia	36	153,947	23.4	10.0
Romania	31	NA	NA	64.0
Finland	30	193,967	15.5	3.6
Slovenia	30	155,458	19.3	4.1
Cyprus	26	75,594	34.4	5.7
Luxembourg	25	109,809	22.8	5.9
Lithuania	13	115,503	11.3	29.0
Iceland	9	27,593	32.6	2.8
Slovakia	8	55,306	14.5	3.2
Hungary	5	72,133	6.9	4.6
Bulgaria	2	4,192	47.7	19.0
Croatia	1	9,438	10.6	6.6
Total	9,116			

IR: incidence rate; NA: not available; TB: tuberculosis; WHO: World Health Organization.

^a^ From the 10 highest ranked countries of origin in terms of number of notified TB cases among foreign-born individuals in the year 2020.

^b^ Incidence rate is reported per 100,000 population in the year 2020.

**Figure 1:**
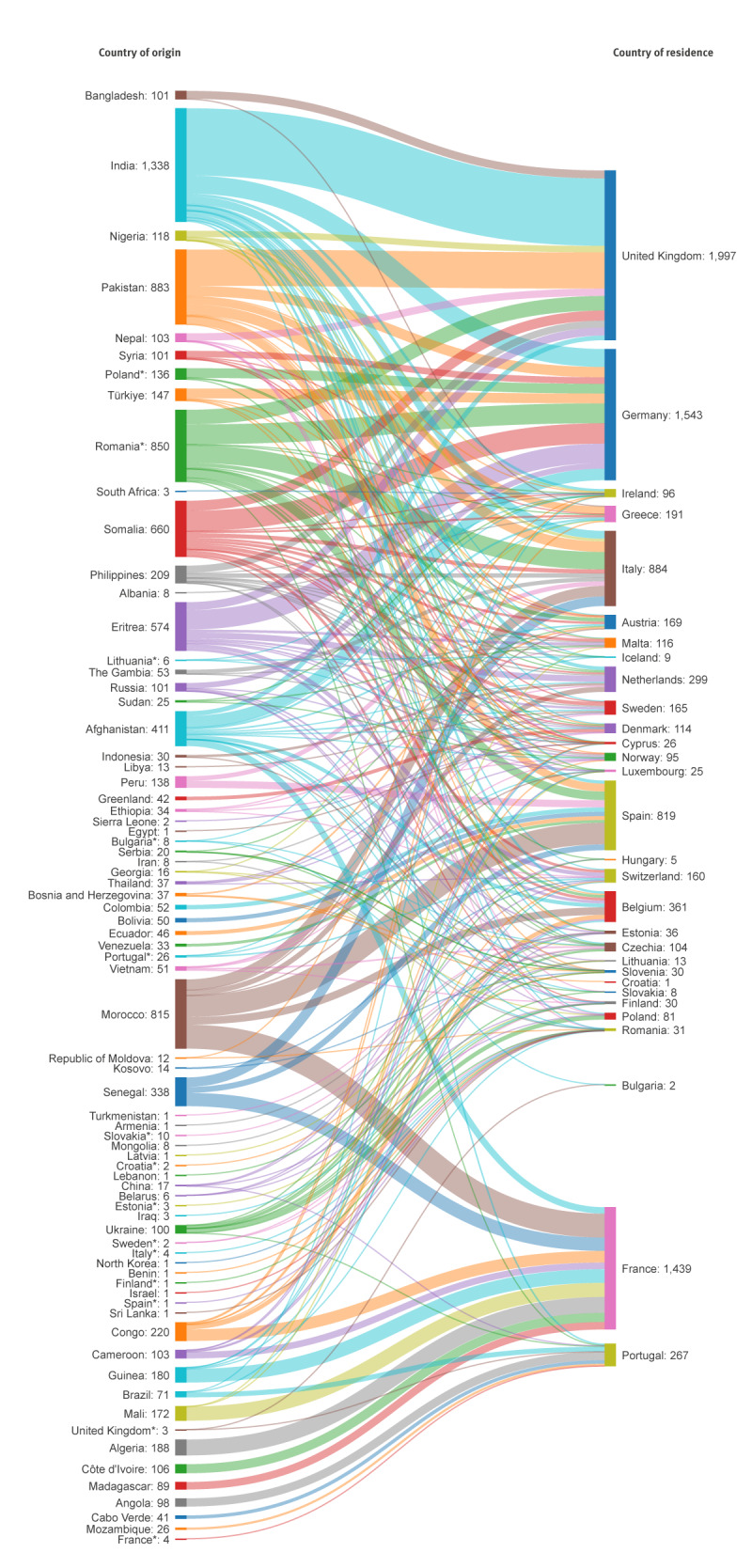
Countries of origin of foreign-born tuberculosis cases residing in European Union countries and Iceland, Norway, Switzerland and the United Kingdom, 2020

Migration between the country of origin and country of residence is shown in Figure 1. Eight countries of origin accounted for two thirds (64.4%) of the 9,116 TB cases in our sample: India (14.7%), Pakistan (9.7%), Romania (9.3%), Morocco (8.9%), Somalia (7.2%), Eritrea (6.3%), Afghanistan (4.5%) and Senegal (3.7%). All other countries accounted for less than 5% of all foreign-born TB cases and less than 500 cases each. In some countries of residence, we highlight specific migratory patterns within their top 10 countries of origin: foreign-born TB cases in the UK were often from Bangladesh, India or Pakistan. Similarly, the main migration pattern of TB cases from Bolivia, Colombia, Ecuador and Venezuela was towards Spain, and TB cases from Angola, Cabo Verde and Mozambique were mostly identified in Portugal.

Countries that appear among the countries of origin and the countries of residence are marked with an asterisk.

**Figure 2:**
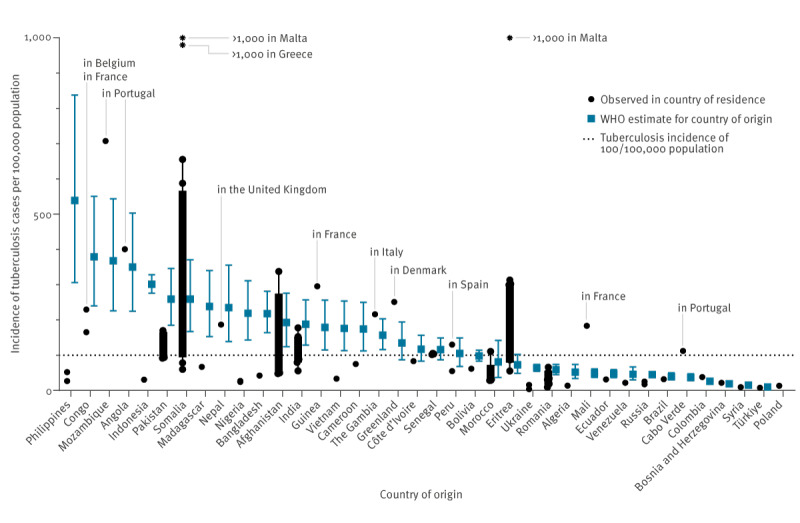
Observed incidence rate of tuberculosis per 100,000 foreign-born individuals per country of residence vs incidence in their country of origin as reported by the WHO based on estimated data, European Union countries and Iceland, Norway, Switzerland and the United Kingdom, 2020

The TB IR calculation included 85 out of 267 populations of foreign-born individuals (Figure 2) accounting for 8,078 of 9,116 patients (88.2%) from 38 countries of origin. A total of 38 of 85 (44.0%) populations had a TB IR of more than 100 per 100,000, 20 (23.5%) had an IR of more than 200 per 100,000 and seven (8.2%) of more than 500 per 100,000. Individuals of Eritrean origin living in Malta and those of Somali origin living in Malta and Greece had the highest TB IR with more than 1,000 per 100,000 population. The lowest IR (3.6/100,000) was found among Ukrainians living in Poland. Among the countries of origin of low TB burden according to WHO estimates (with a WHO-estimated TB IR of less than 100/100,000), there were four countries where foreign-born populations exceeded the TB IR of 100 per 100,000 in their country of residence: (i) people of Moroccan origin living in Belgium (89/80,579) (observed IR: 110.5/100,000, WHO estimated IR: 98.0/100,000); (ii) people of Malian origin living in France (166/90,537) (observed IR: 183.4/100,000, WHO estimated IR: 52.0/100,000); (iii) people from Cabo Verde living in Portugal (41/36,609) (observed IR: 112/100,000, WHO estimated IR: 39.0/100,000). Tuberculosis IR in individuals of Eritrean origin (WHO estimated IR: 81/100,000) was higher than 100 per 100,000 population in all countries of residence except for Norway and Sweden (see below).

WHO: World Health Organization.

Each dot represents incidence in foreign-born residents from one country of origin in one country of residence. Box: interquartile range. Whiskers: min and max.

Each square represents the estimated tuberculosis incidence in a country of origin for 2020. Error bars represent the uncertainty intervals.

The figure is sorted according to the WHO incidence with estimation interval according to a WHO report for 2020.

Incidence by country of origin is only reported if there were at least 20 tuberculosis cases and 100 foreign-born individuals in the country of residence.

Importantly, there were also nine countries of origin of high TB incidence (WHO-estimated IR of more than 100/100,000) where migrant populations had a notably lower TB IR of less than 100 per 100,000 in the country of residence (alphabetical order): Bangladesh, Bolivia, Cameroon, Côte d’Ivoire, Indonesia, Madagascar, Nigeria, Philippines and Vietnam. A comparison of TB IR in foreign-born populations and TB notification rate instead of estimated TB IR is shown in Supplementary Figure S1.

**Figure 3:**
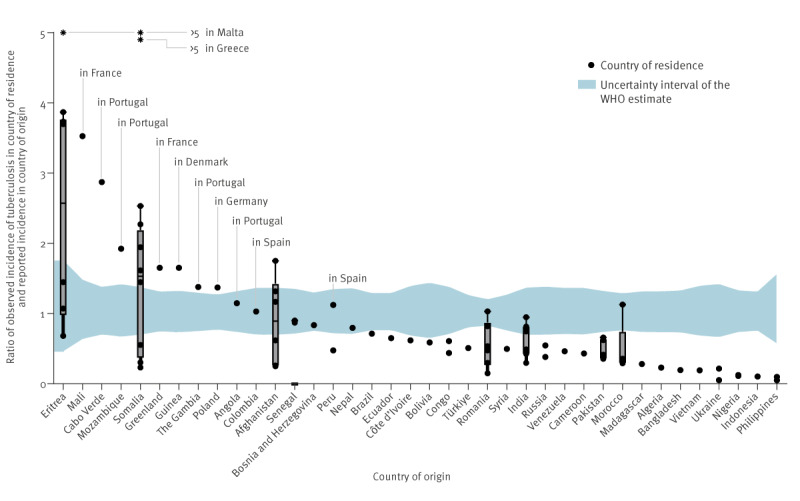
Incidence rate ratio of tuberculosis in country of residence and incidence rate in country of origin as reported by the WHO based on estimated data, European Union countries and Iceland, Norway, Switzerland and the United Kingdom, 2020

Figure 3 shows the TB IRR, i.e. the TB IR in the country of residence divided by the WHO estimated TB IR in the country of origin. Of the 85 included foreign-born populations from 38 countries of origin, 19 (22.4%) from 10 countries had higher TB IRs than in the country of origin according to the WHO [5]. Eighteen populations (21.1%) from 11 countries of origin were within the WHO-estimated IR and its interval of uncertainty. For 48 populations (56.5%) from 25 countries of origin, TB IR in the country of residence was below the WHO estimate. The TB IR was below the WHO estimates for all observed populations for 23 of 37 countries of origin. IRR was highest in populations of Eritrean and Somali origin in Malta and of Somali origin in Greece (IRR > 5), followed by people of Eritrean origin in the UK (IRR=3.9), Germany (IRR=3.7) and the Netherlands (IRR=3.7), people of Malian origin in France (IRR=3.5) and from Cabo Verde in Portugal (IRR=2.9). When the TB IRR was calculated based on notified cases, 46 populations (54.1%) were above and 39 (45.9%) below the IR in the country of origin (Supplementary Figure S2).

Every dot represents one country of residence. Sorted by median ratio. Boxes: interquartile range (IQR), whiskers: min and max.

Incidence rate ratios (IRR) presented above the line (Y = 1) have a higher incidence in the country of residence than in country of origin and IRRs presented below the line have a lower incidence in the country of residence than in country of origin.

Incidence rate by country of origin was only reported if there were at least 20 TB cases and 100 foreign-born individuals in the country of residence.

**Figure 4:**
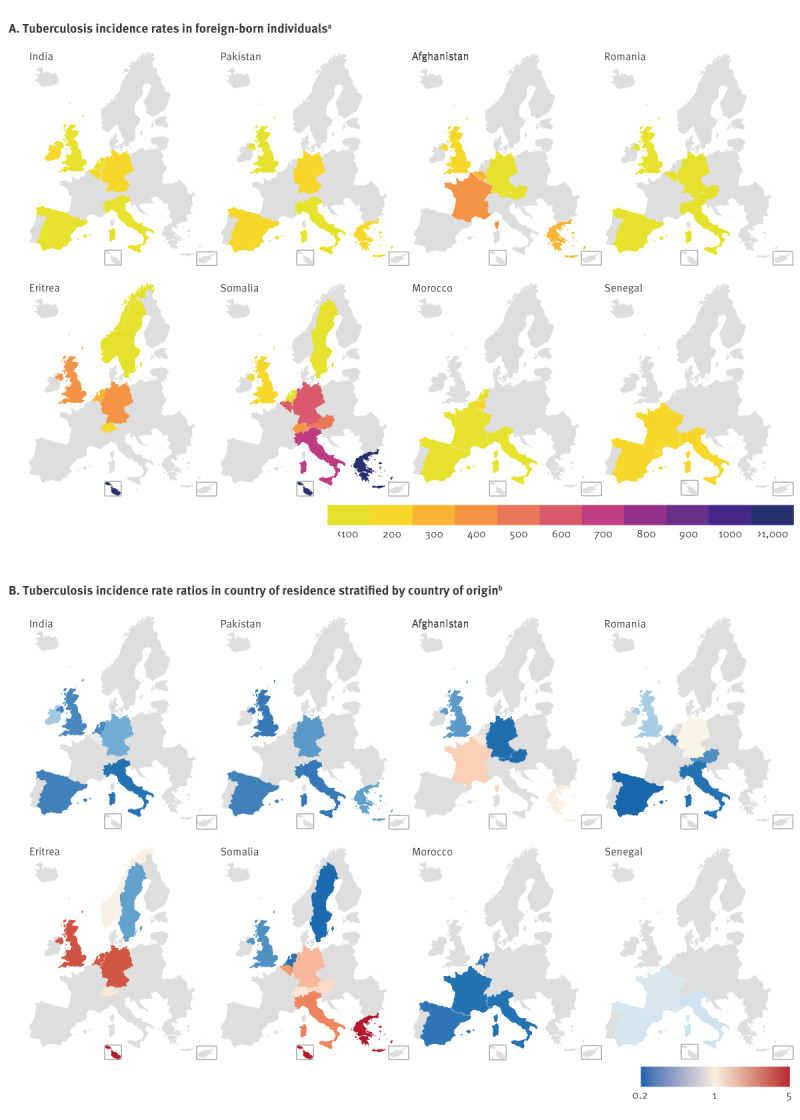
Tuberculosis incidence, European Union, Iceland, Norway, Switzerland and United Kingdom, 2020

^a^ Observed and WHO-estimated TB incidence rate (IR) in foreign-born individuals.

^b^ Tuberculosis IR among foreign-born residents in their country of residence divided by the WHO-estimated TB IR in their country of origin using 2020 data.

The distribution of TB IRs and IRRs in foreign-born populations among countries of residence is shown in Figure 4 for the most common countries of origin of TB cases (alphabetical order): Afghanistan, Eritrea, India, Morocco, Pakistan, Romania, Senegal and Somalia. High tuberculosis IRs (Figure 4A) were observed among populations of Afghani, Eritrean and Somali origin. The population of Afghani origin had a high IR of 337.5 per 100,000 in France, 254.0 per 100,000 in Belgium and 224.8 per 100,000 in Greece, while there was a notably lower IR in the UK (119.1/100,000), Germany (50.0/100,000) and Austria (47.7/100,000) compared with an IR of 193 per 100,000 in Afghanistan. Incidence rates among people of Eritrean origin were highest in Malta (> 1,000/100,000), the UK (313.3/100,000), Germany (302.4/100,000) and the Netherlands (299.4/100,000) compared with 81 per 100,000 in Eritrea. In contrast, TB IRs were below 100/100,000 in Sweden for populations of both Somali and Eritrean origin. People of Somali origin exhibited very high TB IRs in Greece and Malta (more than 1,000/100,000), Italy (655.4/100,000), Belgium (587.8/100,000), Germany (503.2/100,000), Austria (417.7/100,000) and Switzerland (374.4/100,000), while the IR in Somalia was 259 per 100,000 population. Incidence rates in populations from India, Morocco, Pakistan, Romania and Senegal did not surpass 200 per 100,000 in any population or country of residence.

Incidence rate is reported per 100,000 population.

Incidence rate by country of origin was only reported if there were at least 20 TB cases and 100 foreign-born individuals in the country of residence.

The figure has been produced with datawrapper.de

The TB IRR of people originating from Afghanistan in France was 1.7, i.e. the IR was 1.7-fold higher than in Afghanistan (Figure 4B). People of Eritrean origin showed the highest IRRs in Germany, Malta, the Netherlands and the UK (see above) and an IRR of 1.4 in Switzerland. People from Somalia had IRRs of > 5, 2.5, 2.3, 1.9, 1.8 and 1.4 in Greece, Malta, Italy, Belgium, Germany, Austria and Switzerland, respectively. Notably, the IR among people of Somali origin in the UK and both of Eritrean and Somali origin in Sweden was below the WHO-estimated IR range (0.6, 0.7 and 0.2-fold, respectively).

The Bayesian analysis has shown that mean RR of TB in countries of residence varied between 0.34 for Sweden (95% CrI: 0.23–0.46) and 21.0 for Malta (13.2–29.5). The mean RR of TB in countries of origin varied between 0.11 for Albania (0.05–0.17) and 20.5 for Libya (8.79–34.1) (see Supplementary Tables S4 and S5 for details).

## Discussion

In this survey we evaluated TB IRs in foreign-born individuals and compared the relative risk of TB in their country of residence with their country of origin. We identified populations of foreign-born individuals with a high risk of TB, higher than the WHO-estimated risk in their country of origin. To achieve TB elimination in Europe, we conclude that preventative TB strategies must address TB IRs in foreign-born individuals in their country of residence instead of relying on WHO-reported TB IRs in their country of origin only.

Overall, most foreign-born TB patients in Europe originated from (in descending order of burden) India, Romania, Morocco, Pakistan, Somalia, Eritrea, Afghanistan, and Senegal. Reasons for the strong representation of these countries of origin among foreign-born individuals with TB are multifactorial but include historical ties, language, pre-existing communities, demand for workers in certain sectors, international trade and/or migration policies [16], but also migration due to socioeconomic situation, civil unrest/war, climate change and natural disasters. These patterns become evident and are highlighted by migrant flows between countries of origin and countries of residence.

There was substantial heterogeneity in TB IRs among foreign-born individuals throughout the surveyed countries. In many European countries of residence we observed high TB IRs in people from Afghanistan, Eritrea and Somalia, areas with war or conflict situations that facilitate the spread and hamper the prevention and control of TB. However, we also observed high IRs in specific populations such as people from Mozambique residing in Portugal. Previous research has shown that migration from outside the EU/EEA contributes markedly to the TB burden in the EU/EEA [17].

For almost 80% of the populations included in this dataset, the TB IR in the country of residence was not aligned with the WHO-estimated IR in the country of origin. In fact, 60% of populations had an IR below the WHO-estimated IR in the country of origin and 20% had a higher IR than estimated by the WHO for their country of origin.

Possible explanations for lower TB IRs in countries of residence could be lower risk of TB transmission in the country of residence, higher socioeconomic status of the migrants, long time passed since migration [18,19] and a long time since exposure to TB [20]. Effective past screening policies, for example systematic X-ray screening, might also contribute to decreased incidence today. Low TB IRs in Somalian populations in Sweden illustrate this phenomenon. It results from a combination of factors, including longer residency in Sweden for the majority of Somalian individuals and the implementation of effective policies. These policies encompass free TB and latent TB testing for all asylum seekers arriving from Somalia, as well as the administration of BCG vaccinations to children in families with ties to Somalia. These measures have been in place since 2008, with minor modifications in various years and provinces, as stipulated by the Swedish law (SFS nr: 2008:344) [21].

Possible explanations for higher TB IRs in countries of residence are routes of migration that are associated with a higher risk of transmission (e.g. residing in overcrowded refugee camps or travelling in crowded vehicles) as well as post-migration transmission (e.g. residing in refugee centres), short time since exposure or arrival, effective TB screening policies, lower socioeconomic status in their country of origin and a population structure that mostly consist of young men. High IRs in, Afghani, Eritrean and Somalian populations in Malta and/or Greece may be examples of these factors.

People of Eritrean and Somalian origin accounted for more than half of the populations with higher TB IRs in the country of residence than the country of origin. Additionally, for Eritrea in particular, the WHO estimates of 81 cases per 100,000 population are likely underestimated.

The RR of being notified with TB in a country of residence was highest in Malta. This could relate to a high number of migrants arriving in this country, sometimes temporarily. Malta often represents the first contact of non-European populations with the European health system. Generally, there is an increased risk of TB exposure or disease activation during migration, in addition to the risk of abuse, human rights violations [22,23]. Moreover, foreign-born individuals often seek care only at an advanced stage of TB disease [3] and there can be considerable delay in accessing diagnostic services [24].

Our results suggest that the TB IR in the country of origin should not be the only indicator when deciding on active case finding or TB prevention policies for foreign-born populations [25]. Introducing a universal screening threshold for foreign-born individuals based on the TB IR in the country of origin, e.g. at an estimated incidence rate of 100 per 100,000 population in the country of origin, may not be an effective screening method for all European countries. A study conducted in Germany found that the difference in observed yield of TB screening among asylum seekers from different countries of origin decreased gradually when higher cut-offs of WHO-estimated TB IRs were used [26]. In contrast with screening depending on a specific TB incidence in the country of origin, future strategies for TB prevention and early case finding on the path towards TB elimination should be guided by regularly collected country-specific data on populations at risk of developing TB.

Our study has several limitations. Given that individual patient data including age and year of arrival were not available in our data sources, it was not possible to investigate links with population characteristics. Data on age and sex would have been available through the ECDC European Surveillance System (TESSy) database. However, data on the time of incident TB after arrival to the respective country in the EU/EEA are not. Future prospective studies would benefit from including individual patient data to inform screening policies. We only considered notified foreign-born TB patients and only the total number of documented foreign-born individuals in a country of residence. Some TB patients included in our data could be among undocumented foreign-born individuals. Moreover, TB patients may remain undetected and 5–30% of foreign-born migrants may not be documented in their country of residence [27]. It is suggested that the burden of tuberculosis may be 10% higher in undocumented migrants [22,27], while others may have moved and may have been notified in two or more countries in 2020. There was also substantial variation in the country of origin TB IRs based on estimated numbers or notified numbers of cases, reflecting variations in the TB detection gap. Factors that partly drive TB IRs, i.e. risk of transmission during and post migration, time since exposure and/or arrival, tuberculosis-screening policies in the country of residence, socioeconomic status in the country of origin and the reason for migration as well as demographic factors such as age and sex were not available for most surveyed countries. Tuberculosis reactivation rates post-migration are likely dependent on both time from exposure/migration and age [18,20]. Moreover, different European countries’ screening policies certainly impact TB detection and prevention. During the period 2011–2022, the annual average decline in TB IRs in the EU/EAA was 5.2%, with 6.2% achieved between 2019 and 2020 [4]. While it can be assumed that the observed decrease in TB incidence between 2019 and 2020 was partly affected by a decrease in migration to the EU/EEA and decreased TB notifications due to COVID-19 restrictions in 2020 [5], our data are highly relevant to inform TB control strategies for the next decade. We only considered data from the 10 countries of origin with the highest number of foreign-born TB cases in each country of residence to find a balance between a broad representation of all countries in the region and the aim to capture foreign-born individuals at highest risk for TB in European countries of residence. This approach may have led to underrepresentation of foreign-born individuals from countries with a lower number of TB cases in the included countries. Finally, we primarily based our analysis on estimated IRs in countries of origin as reported to the WHO because these data are predominantly available to policymakers. It is possible that for some countries, e.g. Eritrea, where the incidence estimates are low for the region while incidence is high in Eritrean migrants, WHO estimates of TB IRs are inaccurate, leading to incorrect guidance for TB screening.

Despite these limitations, our research using comprehensive latest available data from countries in the European Union and Iceland, Norway, Switzerland and the UK supports national solutions based on recent risk analyses to target TB prevention and case finding strategies among foreign-born individuals. Social determinants of health are known to be strong risk factors for progression towards active TB. Interventions aimed at improving socioeconomic conditions in foreign-born populations could prevent new TB cases.

## Conclusion

The burden of TB among foreign-born individuals is highly diverse and heterogeneously distributed across Europe. A high TB IR in the country of origin is often not aligned with the risk of TB in foreign-born populations living in Europe. Therefore, this should not be the only indicator used to inform guidelines for active case finding and TB prevention in EU countries and Iceland, Norway, Switzerland and the UK. Curative and preventative TB control and elimination strategies should be informed by regularly collected country-specific data to ensure that changing epidemiology and associated risks are addressed.

## Supplementary Data

478000 Supplementary Material
