# Supplementary material for: Tuberculosis incidence in foreign-born people residing in European countries in 2020
Source: Euro Surveill. 2023 Oct 19;28(42):2300051. doi: 10.2807/1560-7917.ES.2023.28.42.2300051 (PMC10588305; doi:10.2807/1560-7917.ES.2023.28.42.2300051)
Supplement: Supplementary Material [file 23-00051_LANGE_Supplement.pdf]

Supplemental File

This supplementary material is hosted by *Eurosurveillance* as supporting information alongside the article **Tuberculosis incidence in foreign-born people residing in European countries in 2020**, on behalf of the authors, who remain responsible for the accuracy and appropriateness of the content. The same standards for ethics, copyright, attributions and permissions as for the article apply. Supplements are not edited by *Eurosurveillance* and the journal is not responsible for the maintenance of any links or email addresses provided therein

Table S1. Notified tuberculosis cases among foreign-born individuals from 10 countries with the highest number of tuberculosis cases in countries of the EU, Iceland, Norway, Switzerland and UK in 2020.

country of residence	countries of origin, number of TB cases, number of foreign-born individuals from this country of origin										
	1	2	3	4	5	6	7	8	9	10	Total
UK	India<br>793<br>896000	Pakistan<br>428<br>456000	Romania<br>172<br>329000	Somalia<br>117<br>82000	Eritrea<br>94<br>30000	Bangladesh<br>94<br>223000	Nepal<br>86<br>46000	Philippines<br>82<br>158000	Nigeria<br>75<br>312000	Afghanistan<br>56<br>47000	2211<br>3718000
Germany	Somalia<br>239<br>47495	Romania<br>234<br>355343	Eritrea<br>229<br>75735	India<br>211<br>150840	Afghanistan<br>136<br>271805	Pakistan<br>121<br>75355	Turkey<br>116<br>1527118	Poland<br>114<br>866690	Syria<br>77<br>818460	Russia<br>66<br>263300	1543<br>4452141
France	Morocco<br>282<br>992455	Algeria<br>188<br>1389899	Mali<br>166<br>90537	Guinea<br>161<br>54493	Senegal<br>156<br>148275	Congo<br>137<br>82891	Côte d'Ivoire<br>106<br>127419	Madagascar<br>89<br>133327	Cameroon<br>78<br>104253	Afghanistan<br>76<br>22519	1439<br>3146068
Italy	Romania<br>204<br>1076412	Pakistan<br>124<br>135520	Morocco<br>124<br>428947	Senegal<br>113<br>111092	India<br>92<br>165512	Peru<br>53<br>96546	Somalia<br>50<br>7629	Gambia<br>48<br>22213	Philippines<br>44<br>165443	Nigeria<br>32<br>119089	884<br>2328403
Spain	Morocco<br>253<br>811530	Romania<br>102<br>1079726	Pakistan<br>93<br>87251	Peru<br>85<br>65331	Senegal<br>69<br>66560	Colombia<br>52<br>136762	Bolivia<br>50<br>81196	Ecuador<br>46<br>147974	India<br>36<br>45178	Venezuela<br>33<br>152017	819<br>2673525
Belgium	Morocco<br>89<br>80579	Congo<br>49<br>21357	Afghanistan<br>45<br>17717	Eritrea<br>34	Somalia<br>34<br>5784	Romania<br>32<br>105358	India<br>28<br>15747	Cameroon<br>19<br>13201	Guinea<br>16<br>9948	Turkey<br>15<br>37492	361<br>307183
Netherlands	Eritrea<br>74<br>24712	Morocco<br>61<br>174664	India<br>42<br>45759	Indonesia<br>29<br>95178	Somalia<br>20<br>25543	Ethiopia<br>16<br>18379	Philippines<br>16<br>15710	Poland<br>15<br>159055	Romania<br>14<br>34136	Afghanistan<br>12<br>37319	299<br>630455
Portugal	Angola<br>98<br>24449	Brazil<br>59<br>183993	Cabo Verde<br>41<br>36609	Mozambique<br>26<br>3675	India<br>18<br>24550	Romania<br>8<br>30052	Ukraine<br>8<br>28159	France<br>4<br>24935	China<br>3<br>26074	UK<br>2<br>46238	267<br>428734
Greece	Pakistan<br>85<br>50000	Afghanistan<br>24<br>10675	Somalia<br>24<br>1524	Congo<br>16<br>1769	Albania<br>8<br>500000	Georgia<br>8<br>27000	Romania<br>8<br>46000	Bangladesh<br>7<br>11000	Syria<br>7<br>10000	Bulgaria<br>4<br>75000	205<br>745905
Austria	Romania<br>45<br>131824	Somalia<br>28<br>6703	Afghanistan<br>21<br>44002	Bosnia and Herzegovina<br>21<br>96990	Turkey<br>14<br>117580	Serbia<br>13<br>121990	India<br>9<br>9692	Syria<br>8<br>55372	Pakistan<br>5<br>4112	Morocco<br>5<br>1500	171<br>601663
Sweden	Somalia<br>42<br>70184	Eritrea<br>26<br>47156	Afghanistan<br>19<br>60858	Philippines<br>16<br>15640	Thailand<br>15<br>44339	Libya<br>13<br>3810	India<br>10<br>42790	Vietnam<br>9<br>21126	Syria<br>8<br>193594	Iran<br>7<br>81301	165<br>580798

Switzerland	Eritrea<br>48<br>40969	Somalia<br>31<br>8281	Portugal<br>19<br>257691	China<br>11<br>19886	India<br>11<br>16085	Kosovo<br>11<br>113660	Thailand<br>8<br>9524	Ethiopia<br>7<br>4208	Brazil<br>7<br>22384	Congo<br>7<br>5992	167<br>517513
Malta	Somalia<br>33<br>481	Eritrea<br>31<br>715	Sudan<br>17	India<br>8	Philippines<br>8	Mali<br>5	Ethiopia<br>4	Gambia<br>4	Nepal<br>3	Nigeria<br>3	133<br>1196
Denmark	Greenland<br>42<br>16734	Somalia<br>14<br>11228	Eritrea<br>9<br>5908	Pakistan<br>9<br>14478	Romania<br>8<br>29576	India<br>8<br>12852	Philippines<br>7<br>10665	Thailand<br>7<br>11949	Vietnam<br>6<br>15953	Afghanistan<br>4<br>13883	114<br>143226
Czech Republic	Ukraine<br>26<br>165654	Vietnam<br>21<br>62884	India<br>17<br>5813	Slovakia<br>10<br>124544	Mongolia<br>8<br>10141	Romania<br>7<br>18396	Poland<br>6<br>20733	Georgia<br>3<br>1724	Bulgaria<br>3<br>17917	Philippines<br>3<br>2631	104<br>430437
Ireland	India<br>32<br>20969	Philippines<br>13<br>14725	Pakistan<br>12<br>12891	Romania<br>6<br>28702	Brazil<br>5<br>15796	Somalia<br>4<br>1500	Nigeria<br>4<br>16569	Lithuania<br>4<br>33344	Congo<br>4<br>2722	Afghanistan<br>3<br>1729	96<br>165080
Norway	Eritrea<br>20<br>23075	Somalia<br>15<br>28554	Philippines<br>14<br>23280	Afghanistan<br>9<br>17293	Ethiopia<br>7<br>8685	Vietnam<br>7<br>14329	Thailand<br>7<br>21097	Congo<br>6<br>3444	Sudan<br>5<br>4873	Pakistan<br>5<br>21097	95<br>165727
Poland	Ukraine<br>49<br>1351418	India<br>14<br>33107	Nepal<br>7<br>9175	Georgia<br>5<br>27917	Russia<br>3<br>37030	Belarus<br>3<br>105404	-	-	-	-	81<br>1564051
Estonia	Russia<br>20<br>114489	Ukraine<br>8<br>25676	Belarus<br>2<br>10305	Turkmenistan<br>1<br>336	Armenia<br>1<br>742	Cameroon<br>1<br>114	Pakistan<br>1<br>426	Nigeria<br>1<br>823	India<br>1<br>1036	-	36<br>153947
Romania	Republic of Moldova<br>9<br>23279	Nepal<br>4	Italy<br>4<br>9835	India<br>2	Guinea<br>1	North Korea<br>1	Afghanistan<br>1	Benin<br>1	Bulgaria<br>1	Finland<br>1	31<br>69077
Finland	Vietnam<br>8<br>9559	Somalia<br>8<br>13929	Estonia<br>3<br>50866	Iraq<br>3<br>20392	Russia<br>3<br>63756	China<br>2<br>11459	Sweden<br>1<br>8987	Ukraine<br>1<br>5837	Afghanistan<br>1<br>9182	-	30<br>193967
Slovenia	Bosnia and Herzegovina<br>15<br>73179	Serbia<br>4<br>35820	Kosovo<br>3<br>19577	Croatia<br>2<br>10111	Lebanon<br>1<br>11	Turkey<br>1<br>263	Afghanistan<br>1<br>49	Indonesia<br>1<br>51	Philippines<br>1<br>154	Serbia<br>1<br>16243	30<br>155458
Cyprus	Romania<br>6<br>43827	Cameroon<br>4<br>202	Sierra Leone<br>2<br>21	Nepal<br>2<br>5807	Nigeria<br>2<br>386	Egypt<br>1<br>2347	Guinea<br>1<br>18	India<br>1<br>6022	Lithuania<br>1<br>1784	Philippines<br>1<br>6788	26<br>75594

Luxembourg	Eritrea<br>9<br>1471	Portugal<br>5<br>94335	India<br>4<br>3125	Cameroon<br>1<br>737	Guinea<br>1<br>312	Morocco<br>1<br>1484	Mali<br>1<br>30	Nigeria<br>1<br>313	Romania<br>1<br>6096	Russia<br>1<br>1906	25<br>109809
Lithuania	Russia<br>8<br>48320	Ukraine<br>3<br>25690	Belarus<br>1<br>36003	Latvia<br>1<br>5490	-	-	-	-	-	-	13<br>115503
Iceland	Philippines<br>4<br>2276	Portugal<br>2<br>1034	Lithuania<br>1<br>3291	Poland<br>1<br>20553	India<br>1<br>439	-	-	-	-	-	9<br>27593
Slovakia	Ukraine<br>3<br>38307	Afghanistan<br>2<br>390	Serbia<br>2<br>16604	Nepal<br>1<br>5	-	-	-	-	-	-	8<br>55306
Hungary	Romania<br>3<br>22162	China<br>1<br>19655	Ukraine<br>1<br>30316	-	-	-	-	-	-	-	5<br>72133
Bulgaria	UK<br>1<br>3857	Afghanistan<br>1<br>335	-	-	-	-	-	-	-	-	2<br>4192
Croatia	Bosnia and Herzegovina<br>1<br>9438	-	-	-	-	-	-	-	-	-	1<br>9438

Table S2. Notified tuberculosis cases among foreign-born individuals. For Ireland, Romania, and Cyprus, up to 16 countries were included in the analysis because there was no clear ranking for the 10th country due to an equivalent number of tuberculosis cases.

country of residence	country of origin<br>number of TB cases<br>number of foreign-born individuals from this country of origin					
	11	12	13	14	15	16
Ireland	South Africa<br>3<br>8085	Sudan<br>3<br>1576	Republic of Moldova<br>3<br>6472	-	-	-
Romania	Iran<br>1	Israel<br>1<br>452	Spain<br>1<br>32671	Sri Lanka<br>1	Sweden<br>1<br>402	Ukraine<br>1<br>2438
Cyprus	Congo<br>1<br>88	Gambia<br>1<br>12	Somalia<br>1<br>271	Syria<br>1<br>7560	Turkey<br>1<br>461	-

Table S3: Sources of data on foreign-born patients who developed tuberculosis in 2020 and on the number of foreign-born individuals known to live in a country.

Country	Source of data on foreign-born residents with tuberculosis living in the country	Source of data on number of foreign-born residents living in the country
Austria	EPidemiological notification system (EMS), Ministry of Health, Austria, and Institute for Infection Epidemiology/Agency for Health and Food Security (AGES), Vienna, Austria	Demographic yearbook 2022, Statistik Austria, National Institute for Statistics, Vienna, Austria<br><br>https://www.statistik.at/fileadmin/publications/Demographisches-JB-2020.pdf<br>https://www.statistik.at/fileadmin/pages/407/Bev_Staatsangeh_Bundesl_seit_2002.ods
Belgium	Belgian Tuberculosis Registry 2020 –FARES asbl/VRGT vzw – March 2022, Brussels, Belgium<br><br>https://www.fares.be/tuberculose/publications/rapports-epidemiologiques/fares-registretbc2020_vd-1.pdf	National Institute for Statistics Belgium (https://statbel.fgov.be/en/open-data
Bulgaria	National Program for Prevention and Control of Tuberculosis in Bulgaria	Data on foreign-born population living in Bulgaria in 2020 not available
Croatia	National TB register of Croatia	Croatian Bureau of Statistics, https://podaci.dzs.hr/2021/en/10485
Cyprus	Cyprus National Reference Laboratory for Mycobacteria, Nicosia, Cyprus	Cyprus Civil Registry and Migration Department, Nicosia, Cyprus
Czech-Republic	National TB register of Czech Republic, Prague<br>https://www.uzis.cz/res/f/008358/tbc2020-en.pdf	Bureau of Statistics of the Ministry of Interior of the Czech Republic, Prague<br>https://www.mvcr.cz/clanek/cizinci-s-povolenym-pobytem.aspx?q=Y2hudW09Mw%3d%3d
Denmark	National TB Register for Denmark<br>Statens Serum Institut<br>Data on TB in Denmark available on:<br>https://statistik.ssi.dk/	Statistics Denmark<br>Data on foreign-born residents living in Denmark available on:<br>https://www.dst.dk/en/Statistik/emner/borgere/befolkning/indvandrere-og-efterkommere
Estonia	Estonian Tuberculosis Registry<br>National Institute for Health Development	Statistics Estonia<br>https://andmed.stat.ee/en/stat/rahvastik__rahvastikunaitajad-ja-koosseis__rahvaarv-ja-rahvastiku-koosseis/RV069U

	https://statistika.tai.ee/pxweb/en/Andmebaas/Andmebaas__02Haigestumus__03Tuberkuloos/TB84.px/<br>https://statistika.tai.ee/pxweb/en/Andmebaas/Andmebaas__02Haigestumus__03Tuberkuloos/TB94.px/<br><br>Data for this purpose data was detailed in TB registry individually, because on homesite is published only places of birth Estonia or other country	
Finland	National Infectious Diseases Register (NIDR)<br>Finnish Institute for Health and Welfare<br><br>https://www.thl.fi/ttr/gen/rpt/tilastot.html	Statistics Finland<br>https://statfin.stat.fi/PxWeb/pxweb/en/StatFin/
France	TB mandatory notification/Santé publique France	Institut national de la statistique et des études économiques (Insee)
Germany	Bericht zur Epidemiologie der Tuberkulose<br>in Deutschland für 2020<br>Robert Koch-Institut, Berlin 2021<br><br>https://www.rki.de/DE/Content/InfAZ/T/Tuberkulose/Download/TB2020.pdf?__blob=publicationFile	Statistisches Bundesamt. Bevölkerung und Erwerbstätigkeit.<br>https://www.destatis.de/DE/Themen/Gesellschaft-Umwelt/Bevoelkerung/Migration-Integration/Publikationen/Downloads-Migration/auslaend-bevoelkerung-2010200207004.pdf?__blob=publicationFile
Greece	Tuberculosis registry at the National Public Health Organization (NPHO) of Greece	Combined data from the Ministry of Migration of Greece, the Ministry of Health of Greece, the<br>Ministry of Labour of Greece, the National Statistics Agency of Greece and the Embassies of countries of origin of TB cases
Hungary	TB Surveillance Centre of Hungary<br>Koranyi National Institute for Pulmonology<br>Data published in the national TB situation chapter of the Korányi Bulletin: https://szakmai.koranyi.hu/wp-content/uploads/2021/11/Evkonyv2021.pdf<br><br>in combination with the TB register of Hungary	Hungarian Central Statistical Office<br>https://www.ksh.hu/stadat_files/nep/hu/nep0023.html
Iceland	National registry of infectious diseases of Iceland.	Statistics Iceland<br>https://px.hagstofa.is/pxen/pxweb/en/Ibuar/Ibuar__mannfjoldi__3_bakgrunnur__Faedingarland/MAN12103.px
Ireland	Data extracted from the Irish national TB surveillance data on 30/08/2022 as these data were not published.	2016 denominator data provided by the Central Statistics Office, Ireland:

		Home\Census 2016\Profile 7 - Migration and Diversity\E7050 - Population Usually Resident and Present in the State 2011 to 2016<br>https://data.cso.ie/<br>More recent denominator data are not available broken down by country of birth.
Italy	Italian Ministry of Health, National System of Infectious Disease Notification.	Italian National Institute of Statistics (ISTAT). http://stra-dati.istat.it/
Latvia	Data not available	Data not available
Lithuania	Tuberculosis State information system, Lithuania	Department of Statistics to the Government of the Republic of Lithuania<br><br>https://osp.stat.gov.lt/lietuvos-gyventojai-2020/salies-gyventojai/gyventoju-skaicius-ir-sudetis
Luxemburg	The national digital data base for infectious diseases of Luxemburg	Lu'stat Statistics Luxembourg<br>https://lustat.statec.lu/vis?fs[0]=Th%C3%A8mes%2C1%7CPopulation%20et%20emploi%23B%23%7CEtat%20de%20la%20population%23B1%23&pg=0&fc=Th%C3%A8mes&df[ds]=ds-release&df[id]=DF_B1113&df[ag]=LU1&df[vs]=1.0&pd=2015%2C2022&dq=.A
Malta	National TB database at the Infectious Disease Prevention and Control Unit, Health Promotion and Disease Prevention Directorate, Superintendence of Public Health, Ministry for Health of Malta, La Valetta, Malta.	Data on foreign-born population living in Malta in 2020 not available
Netherlands	'Nederlands Tuberculose Register'<br>National Institute of Public Health and the Environment (RIVM – Cib) of the Netherlands,<br>Centre Infectious Disease Control<br>Epidemiology and Surveillance Unit	Database Statistics Netherlands<br>StatLine (cbs.nl)<br>StatLine - Bevolkingsontwikkeling; migratieachtergrond en generatie (cbs.nl)<br>and<br>StatLine - Asielverzoeken en nareizigers; nationaliteit, geslacht en leeftijd (cbs.nl)
Norway	Norwegian institute of Public Health<br><br>Website: www.fhi.no	Statistics Norway<br><br>Website: www.ssb.no
Poland	National Tuberculosis and Lung Diseases Research Institute, Warsaw, Poland<br>https://www.igichp.edu.pl/diagnostyka/zaklad-epidemiologii-i-organizacji-walki-z-gruzlica/	Demography Database/Current research results/Imigration of the Central Statistical Office Poland:https://demografia.stat.gov.pl/BazaDemografia/Tables.aspx<br><br>and the Demographic Yearbook 2021<br>https://stat.gov.pl/obszary-tematyczne/roczniki-statystyczne/roczniki-statystyczne/rocznik-demograficzny-2021,3,15.html).

Portugal	TB cases among migrants in Portugal: SVIG-TB (sistema de vigilância intrínseco do Programa Nacional de Luta Contra a Tuberculose). https://www.dgs.pt/documentos-e-publicacoes/relatorio-de-vigilancia-e-monitorizacao-da-tuberculose-em-portugal-dados-definitivos-2020-pdf.aspx	Servicio de Estrangeiros de Fronteiras, Baracarena, Portugal<br>https://sefstat.sef.pt/Docs/Rifa2020.pdf
Romania	Electronic TB Register of National TB Programme in Romania	Romanian National Institute for Statistics
Slovakia	National TB Registry of Slovakia	International Office of Migration Slovakia
Slovenia	Tuberculosis Registry of Republic of Slovenia,<br>University Clinic of Respiratory and Allergic Diseases, Golnik<br><br>https://www.klinika-golnik.si/register-tuberkuloze-republike-slovenije	Statistical office; Republic of Slovenia<br><br>https://pxweb.stat.si/sistat/sl/Podrocja/Index/100/prebivalstvo
Spain	The National Network of Epidemiological Surveillance. Epidemiology National Center.	Ministry of inclusion, social security and immigration. Spanish Government
Sweden	Public Health Service of Sweden<br>https://www.folkhalsomyndigheten.se/	Central Statistical Bureau of Sweden.<br>https://www.scb.se/
Switzerland	Obligatorisches Meldesystem Schweiz, Bundesamt für Gesundheit, 3003 Bern	Bevölkerungsstatistik, Bundesamt für Statistik, Neuenburg<br>https://www.bfs.admin.ch/bfs/de/home/statistiken/bevoelkerung/migration-integration/auslaendische-bevoelkerung.assetdetail.18344247.html
UK	UK Enhanced Tuberculosis Surveillance System (ETS), now moving to National TB Surveillance System (NTBS).<br><br>https://www.gov.uk/government/collections/tuberculosis-and-other-mycobacterial-diseases-diagnosis-screening-management-and-data	UK Office for National Statistics<br>https://www.ons.gov.uk/peoplepopulationandcommunity/populationandmigration/internationalmigration<br><br>https://www.statista.com/statistics/283287/net-migration-figures-of-the-united-kingdom-y-on-y

Table S4 Distribution of relative risk for tuberculosis in foreign-born individuals in the European Union, Iceland, Norway Switzerland and the UK by country of residence, estimated using integrated Laplace approximation

Country of residence	Mean RR	SD	95% Credibility Interval
Malta	21.00	4.25	13.20-29.50
Romania	1.93	0.89	0.57-3.70
Iceland	1.88	0.65	0.74-3.17
Greece	1.84	0.32	1.25-2.48
Belgium	1.81	0.30	1.24-2.41
Slovenia	1.63	0.45	0.84-2.53
Estonia	1.46	0.34	0.83-2.15
Germany	1.46	0.23	1.03-1.92
Luxembourg	1.38	0.35	0.74-2.09
Czech Republic	1.35	0.28	0.84-1.91
Slovakia	1.32	0.47	0.50-2.26
Austria	1.28	0.22	0.87-1.73
Ireland	1.11	0.21	0.73-1.52
Croatia	1.02	0.62	0.13-2.23
UK	0.95	0.15	0.67-1.25
Portugal	0.93	0.19	0.58-1.32
Lithuania	0.92	0.29	0.41-1.50
France	0.90	0.15	0.63-1.20
Netherlands	0.85	0.14	0.59-1.14
Switzerland	0.78	0.14	0.53-1.06
Cyprus	0.74	0.18	0.41-1.09
Italy	0.70	0.11	0.49-0.92
Spain	0.68	0.11	0.47-0.90
Denmark	0.64	0.13	0.41-0.89
Norway	0.44	0.08	0.29-0.60
Finland	0.42	0.10	0.24-0.62
Hungary	0.42	0.16	0.15-0.73
Poland	0.36	0.08	0.23-0.52
Sweden	0.34	0.06	0.23-0.46

RR= relative risk, SD= standard deviation

Table S5. Distribution of relative risk of tuberculosis in foreign-born individuals in the European Union, Iceland, Norway Switzerland and the UK by country of origin, estimated using integrated Laplace approximation

Country of origin	Mean RR	SD	95% Credibility Interval
Libya	20.50	6.79	8.56-34.10
Sierra Leone	8.15	4.57	1.21-17.20
Sweden	7.03	6.11	0.23-18.90
Lebanon	5.96	9.29	0.09-20.10
Mali	5.72	0.95	3.94-7.62
Israel	5.46	7.73	0.08-18.10
Cabo Verde	4.46	1.08	2.51-6.63
Eritrea	4.09	0.60	2.95-5.30
Italy	4.08	2.57	0.53-9.13
Egypt	3.36	3.38	0.07-9.75
Iran	3.23	1.16	1.21-5.53
Turkmenistan	3.13	3.04	0.07-8.93
Armenia	3.04	2.92	0.07-8.61
Mozambique	2.99	0.80	1.56-4.58
Gambia	2.91	0.59	1.83-4.09
France	2.75	1.38	0.55-5.48
Sudan	2.75	0.54	1.74-3.84
Slovakia	2.63	0.94	1.00-4.51
Croatia	2.34	1.57	0.20-5.43
Iraq	2.25	1.02	0.57-4.28
Colombia	2.20	0.44	1.39-3.07
Guinea	2.16	0.35	1.50-2.87
Estonia	1.98	1.10	0.31-4.14
Angola	1.82	0.39	1.12-2.59
Senegal	1.65	0.25	1.17-2.15
Ethiopia	1.56	0.35	0.91-2.26
Peru	1.56	0.26	1.08-2.08
Somalia	1.42	0.21	1.03-1.83
Ecuador	1.40	0.29	0.87-1.97
Poland	1.33	0.21	0.93-1.75
Bolivia	1.27	0.25	0.80-1.77
Lithuania	1.27	0.26	0.80-1.79
Nepal	1.17	0.20	0.79-1.58
Kosovo	1.16	0.35	0.54-1.84
UK	1.15	0.67	0.15-2.48
Brazil	1.13	0.24	0.70-1.61
Venezuela	1.01	0.22	0.59-1.45

Côte d'Ivoire	1.00	0.18	0.67-1.36
Thailand	0.93	0.20	0.56-1.33
Serbia	0.89	0.24	0.45-1.37
Bosnia and Herzegovina	0.85	0.21	0.47-1.26
China	0.83	0.23	0.41-1.29
Portugal	0.74	0.19	0.40-1.11
India	0.74	0.11	0.54-0.95
Cameroon	0.73	0.13	0.49-0.99
Sri Lanka	0.73	0.15	0.45-1.04
Congo	0.68	0.11	0.48-0.89
Romania	0.66	0.10	0.48-0.85
Spain	0.63	0.44	0.05-1.49
Pakistan	0.61	0.09	0.44-0.78
Morocco	0.60	0.09	0.43-0.77
Turkey	0.59	0.10	0.41-0.78
Syria	0.58	0.10	0.39-0.78
Georgia	0.57	0.16	0.28-0.88
Republic of Moldova	0.55	0.25	0.15-1.04
Russia	0.51	0.09	0.34-0.69
Afghanistan	0.48	0.07	0.35-0.62
Belarus	0.47	0.19	0.15-0.84
Madagascar	0.46	0.08	0.31-0.63
Bulgaria	0.39	0.14	0.15-0.67
Vietnam	0.38	0.09	0.22-0.56
Algeria	0.38	0.06	0.26-0.50
Bangladesh	0.30	0.05	0.20-0.40
Mongolia	0.24	0.09	0.09-0.41
Ukraine	0.24	0.05	0.15-0.33
Kenya	0.20	0.04	0.12-0.28
Nigeria	0.20	0.03	0.13-0.26
Indonesia	0.19	0.04	0.11-0.28
Philippines	0.16	0.03	0.11-0.21
South Africa	0.15	0.07	0.04-0.28
Albania	0.11	0.04	0.05-0.18

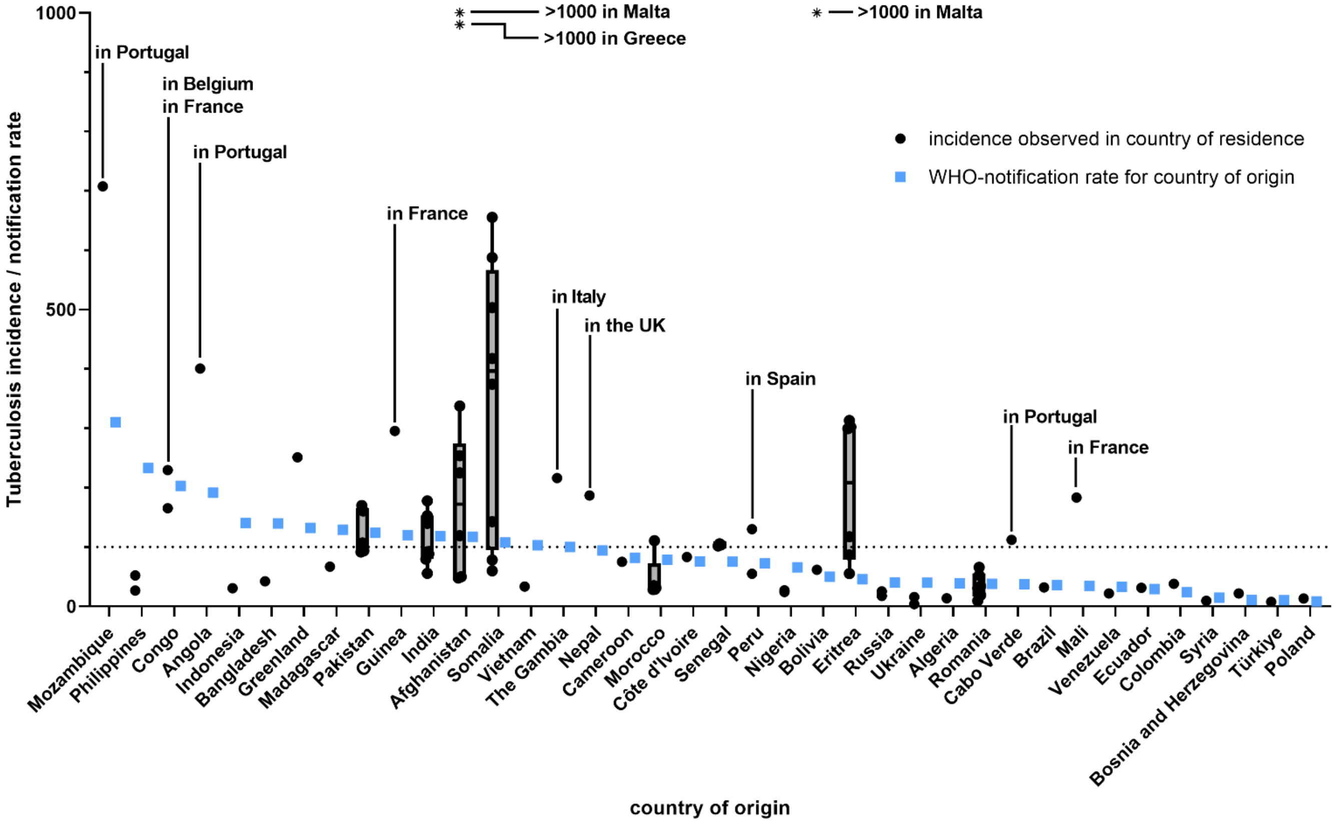

Figure S1. Observed incidence per 100,000 foreign-born individuals from a country of origin versus incidence in the country of origin as reported by WHO based on notified data in the EU, Iceland, Norway, Switzerland and UK for 2020.
Black: Each dot represents incidence in foreign-born residents from one country of origin in one country of residence. Box: interquartile range. Whiskers: min and max. Blue: Each square represents the tuberculosis incidence in a country of origin for 2020. Based on notified data by the WHO. Error bars represent 95% confidence intervals. The figure is sorted according to the tuberculosis incidence based on tuberculosis notification rates according to the WHO for 2020. The dotted line represents a tuberculosis incidence of 100 per 100,000 population. Incidence by country of origin is only reported if there were at least 20 tuberculosis cases and 100 foreign-born individuals in the country of residence.

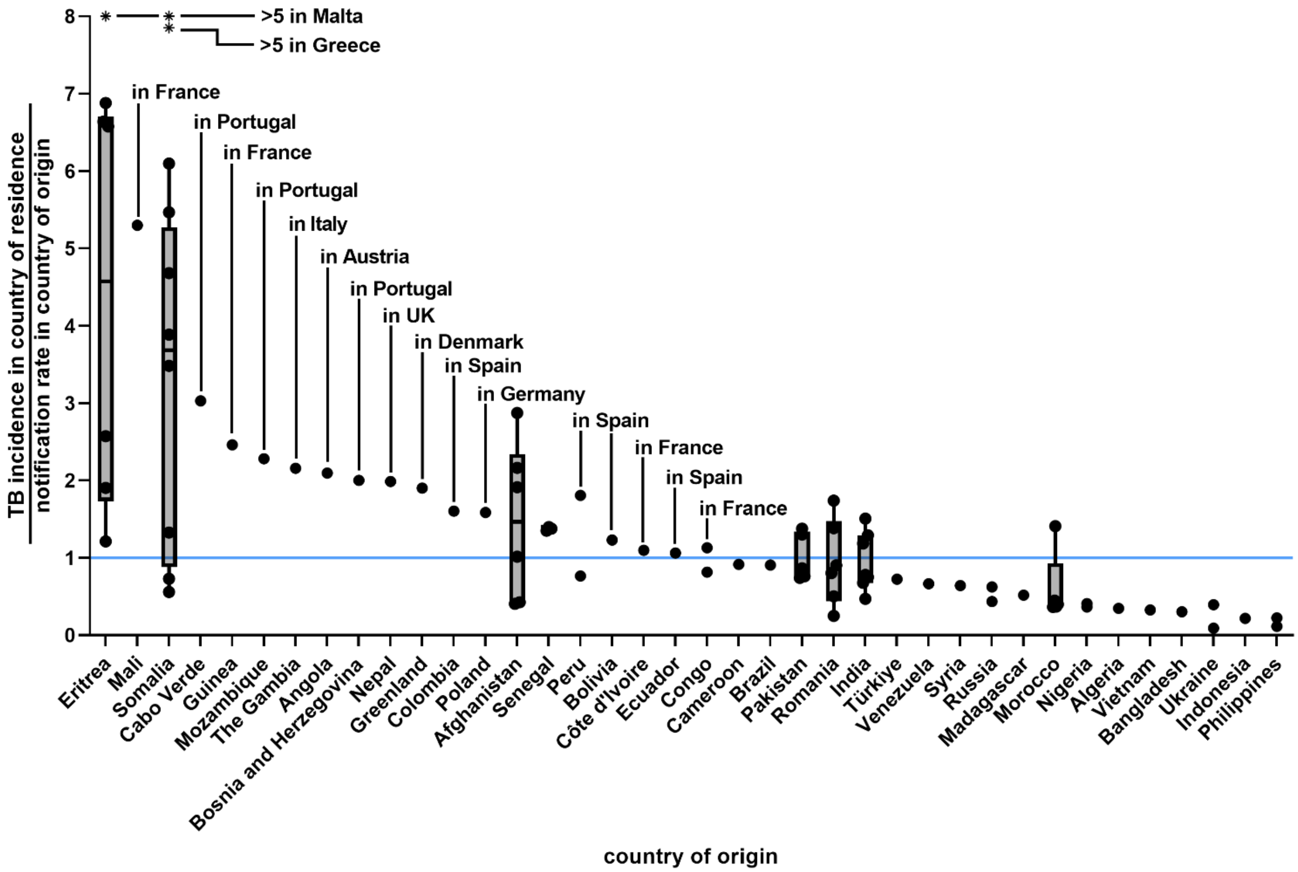

Figure S2. Ratio of observed incidence in country of residence and incidence in country of origin as reported by WHO based on notified data for 2020.

Every dot represents one country of residence. Sorted by median ratio. Box: IQR, whiskers: min and max. Grey area: mean range for tuberculosis incidence based on notified data by WHO for 2020. Ratios presented above the line have a higher incidence in the country of residence than in the country of origin and rations presented below the line have a lower incidence in the country of residence than in the country of origin. Incidence by country of origin was only reported if there were at least 20 tuberculosis cases and 100 foreign-born individuals in the country of residence.

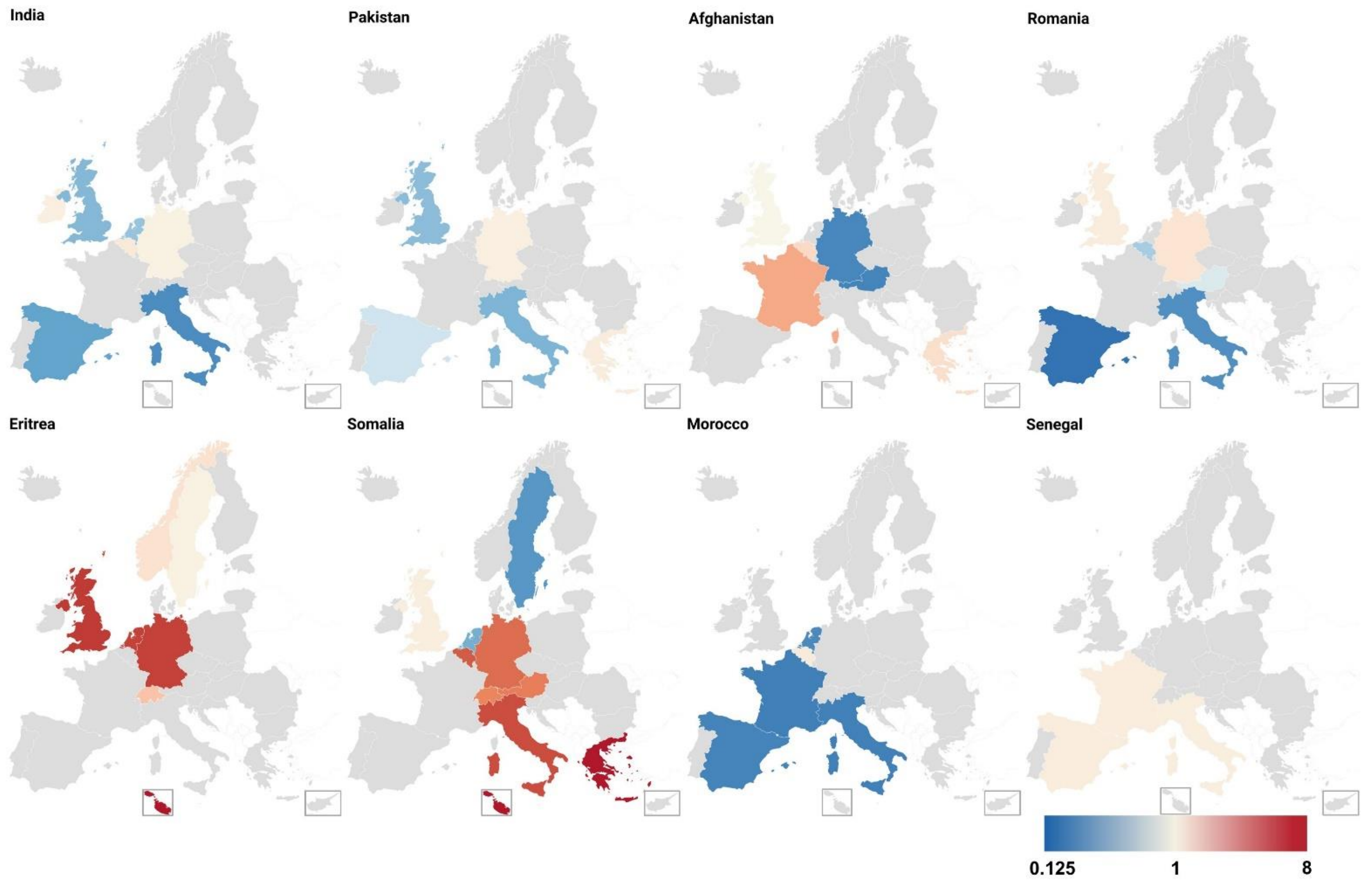

Figure S3. Tuberculosis incidence of foreign-born residents in their country of residence in relation to the tuberculosis incidence in the country of origin based on notified data in the EU, Iceland, Norway, Switzerland and UK for 2020.

Only countries with a ratio of incidence above 1 are represented. Incidence by country of origin was only reported if there were at least 20 tuberculosis cases and 100 foreign-born individuals in the country of residence. The figure has been created with datawrapper.de.
